# Gaucher’s Disease in an Adult Female: A Rare Entity

**DOI:** 10.7759/cureus.17318

**Published:** 2021-08-20

**Authors:** Pankaj K Kannauje, Vinay Pandit, Preetam N Wasnik, Ashish K Gupta, Nanditha Venkatesan

**Affiliations:** 1 General Medicine, All India Institute of Medical Sciences, Raipur, IND; 2 Pathology and Lab Medicine, All India Institute of Medical Sciences, Raipur, IND

**Keywords:** gaucher disease, splenomegaly, enzyme replacement therapy (ert), gaucher cells, bone marrow transplant

## Abstract

Gaucher's disease is a rare inborn error of metabolism with an autosomal recessive pattern of inheritance. With over 26 million births occurring per annum, extrapolation of this figure would give us an estimated burden of 17,000 babies born with lysosomal storage disorder (LSD). Given the large population of India and the high rates of consanguineous marriage that takes place in the subcontinent, LSD might not be as rare as we perceive it to be. We report a rare occurrence of type-1 Gaucher's disease in an adult female patient born of a non-consanguineous marriage, belonging to the tropical area of Chhattisgarh, India where there is a predominance of malaria, thalassemia, and sickling. The diagnosis was challenging in this case since we needed to work out all the differential diagnoses of pancytopenia with hepatomegaly and massive splenomegaly. The key part was her medical history where there was documentation of her elder brother's death due to some mental illness of undiagnosed etiology. Being a difficult time due to coronavirus disease 2019 (‎ COVID-19)‎, we were able to diagnose the patient with a bone marrow biopsy followed by glucocerebrosidase enzyme level suggestive of Gaucher's disease.

## Introduction

Gaucher disease is a type of lysosomal storage disorder (LSD) and has a worldwide incidence between 1:40,000 and 1:86,000, with literature stating a higher prevalence among the Ashkenazi Jews (1:450) [1]. LSD has an estimated incidence of 1:7,000 live births [2].

With the molecular mechanisms being uncovered over the years, glucocerebrosidase was implicated as the enzyme whose deficiency resulted in the development of the disease. However, despite the advances made in understanding the disease, the cost of diagnosis and treatment have impeded the provision of adequate care to the afflicted population. Considered a rare disease, treatment of Gaucher is an arduous task with heavy strain on the family, both emotionally and financially. First-line therapy is intravenous administration of enzyme replacement therapy (ERT) and alternative oral treatment includes substrate reduction therapy (SRT).

In this paper, we report Gaucher's disease in an adult female who was undiagnosed for about six months from the onset of symptoms to disease flare. The catch here was few clinical points that could have diagnosed her earlier.

## Case presentation

A 23-year-old female presented to the outpatient clinic with shortness of breath on exertion, early satiety, and abdominal fullness for nine months. Since she belongs to remote jungles of Chhattisgarh in India which lies along the tropic of cancer, differentials were kept accordingly; her medical history like recurrent fever, jaundice, vaso-occlusive crisis episode, hematemesis, and travel was taken into account and proved non-contributory. On persistent questioning, she confirmed the death of her elder brother with a similar type of illness and mental illness of undiagnosed etiology. 

On examination, the patient was compos mentis with stable vitals. Pallor was noted with hepatomegaly (5 cm) and splenomegaly (10 cm). Differential diagnoses of kala-azar, tropical splenomegaly syndrome, mixed hemoglobinopathy, autoimmune disease, portal hypertension, malignancy, myelofibrosis, and storage disorders, in that order, were made keeping in mind the prevalence of tropical diseases in the country.

On investigation, complete blood count (CBC) showed pancytopenia. Her hemoglobin was 7.1 g/L, platelets 31 x109/L, WBC count of 2.07x109/L, and erythrocyte sedimentation rate (ESR) of 71 mm/hour. Peripheral blood smear showed pancytopenia with moderate aniso-poikilocytosis. Liver and renal function test was normal. Card tests for malaria, human immunodeficiency virus (HIV), hepatitis B and C, Mantoux test were all negative. Upper gastrointestinal endoscopy revealed a normal study. 

**Figure 1:**
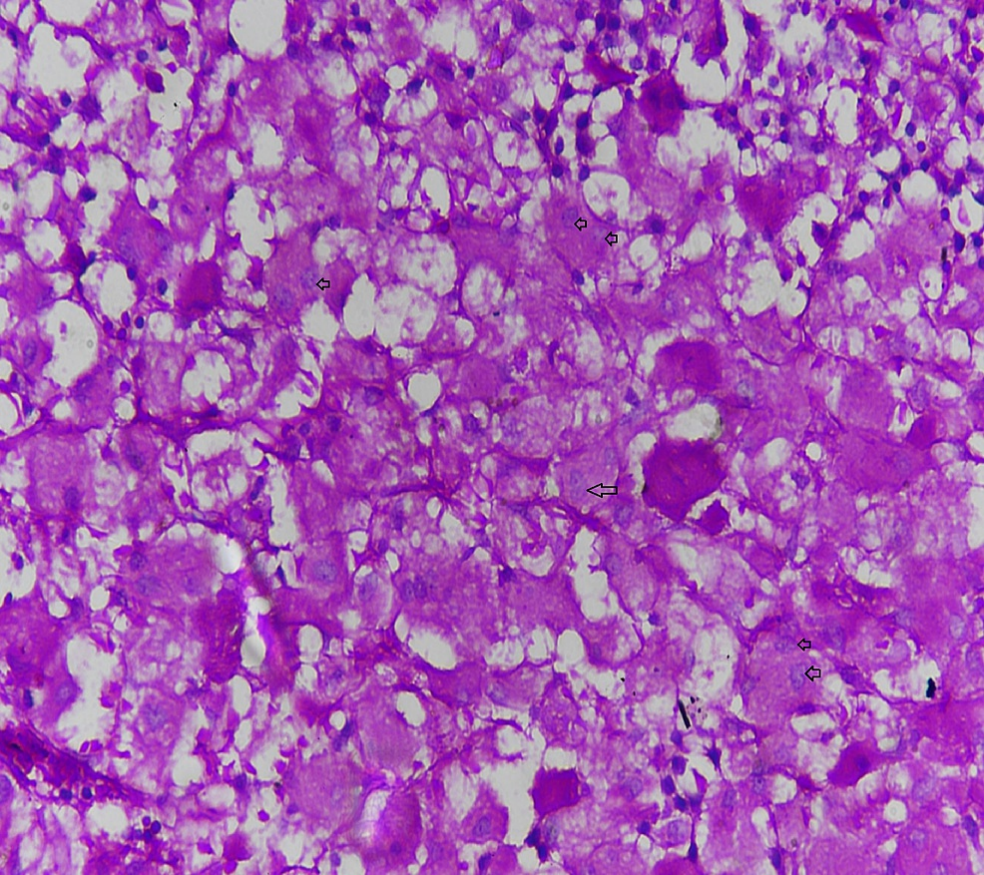
Bone marrow biopsy showing hypercellular bone marrow with marked interstitial to diffuse infiltrates of large periodic acid-Schiff (PAS) stain positive cells with abundant faintly eosinophilic cytoplasm with striations and wrinkles and eccentrically placed, single to multiple nuclei These features are consistent with Gaucher's disease

**Figure 2:**
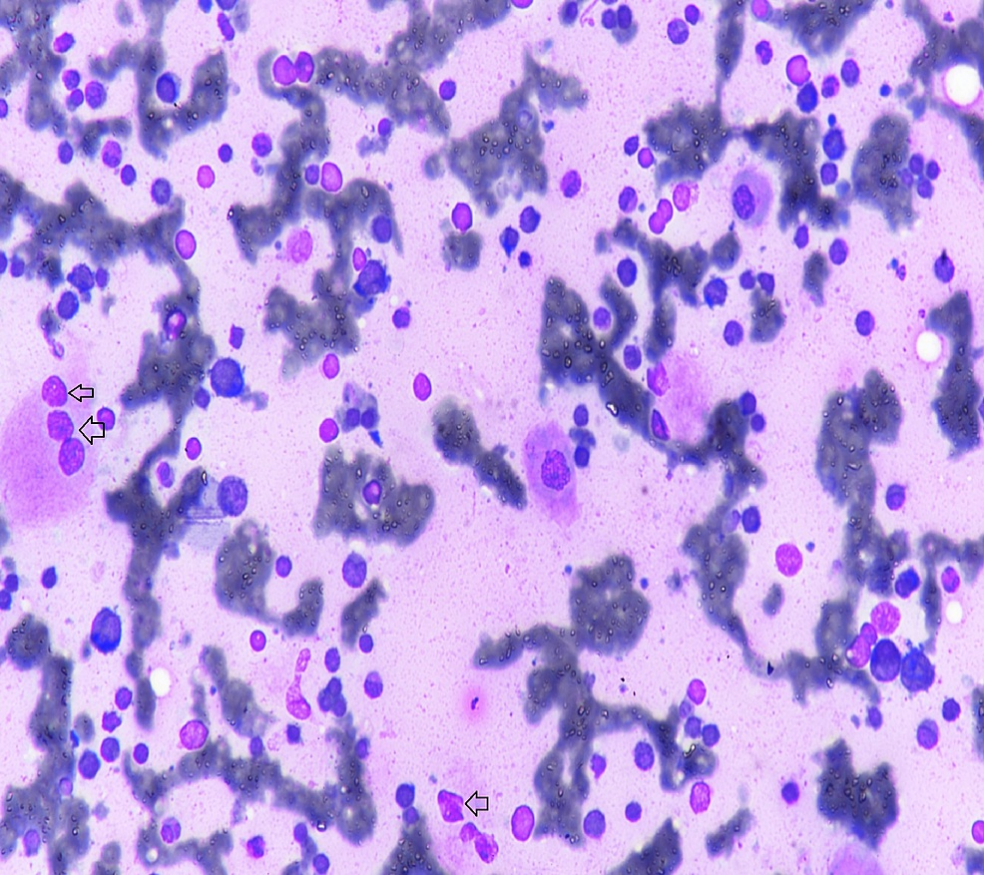
Multiple Gaucher cells (arrows)

Her bone marrow biopsy revealed findings that were consistent with storage disorders and type 1 Gaucher’s disease with enzyme assays was confirmed (Figures 1-2). Enzyme assay of beta-glucosidase recorded a value of 1 nmol/hr/mg. Lab standard values were > 4 for normal activity, 2-4 for carrier states and <2 was labeled as deficient.

Due to her financial constraints, recombinant enzyme treatment could not be initiated immediately. Efforts were made and pharmaceutical companies were approached. In the meantime, there was worsening of pancytopenia and she becomes transfusion-dependent; therefore, splenectomy was planned for which she requested a month’s time. One month later, owing to the lockdown imposed due to the coronavirus disease 2019 (‎ COVID-19)‎ pandemic, the patient was unable to reach the hospital from the confines of her village. Telephonic follow-up revealed that she succumbed to the disease.

## Discussion

Non-hydrolyzation of glucocerebrosides because of mutation in gene encoding for acid beta-glucosidase most commonly GBA1 leads to subsequent infiltration with lipid-laden macrophages of the reticulo-endothelial system [3,4]. In any child presenting with visceromegaly, recurrent infections, bone pain, fatigue, easy bruising, or bleeding, a differential diagnosis of storage disorder is pertinent. On tissue biopsy, these appear as Gaucher cells or foam cells, classically described to have “wrinkled tissue-paper” appearance [5,6].

Broadly there are three clinical subtypes that are delineated based on the extent of neurologic involvement and the age of onset [4,7]. According to literature, the most classic and typical presenting sign of type 1 is splenomegaly [8]. Type 1 Gaucher's disease, known as the chronic, non-neuropathic, adult-type accounts for more than 95% of the cases reported in the literature, making it the most common type. Type 2 is an acute, neuropathic type with infantile-onset where the patient usually succumbs to death before the age of 2 due to complications such as pneumonia. Type 3, also known as Norbottnian type, is a subacute, neuropathic type with juvenile-onset characterized by epileptic seizures that present around 10 years of age and shares a poor prognosis.

Diagnostic delays are not unusual since diagnosis of this disease requires the use of invasive and resource-intensive procedures such as pathological biopsy, molecular diagnosis, and enzyme activity assay [8]. Treatment of Gaucher's disease requires a multi-disciplinary approach involving internal medicine, pediatrics, radiology, pathology, molecular biology, etc. Modalities of treatment include enzyme replacement treatment (ERT), substrate reduction therapy (SRT), bone marrow transplantation, splenectomy, and if needed, blood transfusion [8]. Presently, ERT being a life-long treatment is exorbitantly expensive and economically infeasible in low and middle-income countries. It has proved to be effective in decreasing the size of liver and spleen, ameliorating skeletal abnormalities and hematological disturbances [9]. Second-line therapy is SRT which inhibits glucosylceramide synthase, which catalyzes the first step in the biosynthesis of glucosylceramide, and therefore reduces the biosynthesis of complex glycosphingolipids. In settings where ERT is not feasible, bone marrow transplantation may be offered to a patient once the pros and cons have been enumerated and explained. Splenectomy can lead to infections with encapsulated bacteria.

## Conclusions

Gaucher’s disease has a long course that is cumbersome to diagnose, has variable prognosis depending on the number, type, and extent of organ systems involved, with the additional interplay of social economics. Through our case, we aim to highlight the need to consider storage disorders promptly as an appropriate differential that will aid not only in early diagnosis and treatment but will also have a positive impact on the long-term management and prognosis of the patient. This compounded by a lack of awareness among treating physicians, heavy under-reporting, dearth in tertiary genetic referral centers, and a jarring lack of universal health insurance policy, contribute to the low estimates of prevalence. Since many of these patients travel long distances to avail treatment, the cost of accommodation, travel, repeated hospital visits and, stay depletes the patient’s financial resources. The possibility of a higher prevalence than published report reiterates the pressing need for appropriate and affordable treatment options.
